# SimulFold: Simultaneously Inferring RNA Structures Including Pseudoknots, Alignments, and Trees Using a Bayesian MCMC Framework

**DOI:** 10.1371/journal.pcbi.0030149

**Published:** 2007-08-10

**Authors:** Irmtraud M Meyer, István Miklós

**Affiliations:** 1 UBC Bioinformatics Centre, University of British Columbia, Vancouver, British Columbia, Canada; 2 Department of Computer Science, University of British Columbia, Vancouver, British Columbia, Canada; 3 Alfréd Rényi Institute of Mathematics, Hungarian Academy of Sciences, Budapest, Hungary; 4 Computer and Automation Research Institute (MTA-SZTAKI), Hungarian Academy of Sciences, Budapest, Hungary; 5 eScience Regional Knowledge Centre, Eötvös Loránd University, Budapest, Hungary; University of Queensland, Australia

## Abstract

Computational methods for predicting evolutionarily conserved rather than thermodynamic RNA structures have recently attracted increased interest. These methods are indispensable not only for elucidating the regulatory roles of known RNA transcripts, but also for predicting RNA genes. It has been notoriously difficult to devise them to make the best use of the available data and to predict high-quality RNA structures that may also contain pseudoknots. We introduce a novel theoretical framework for co-estimating an RNA secondary structure including pseudoknots, a multiple sequence alignment, and an evolutionary tree, given several RNA input sequences. We also present an implementation of the framework in a new computer program, called SimulFold, which employs a Bayesian Markov chain Monte Carlo method to sample from the joint posterior distribution of RNA structures, alignments, and trees. We use the new framework to predict RNA structures, and comprehensively evaluate the quality of our predictions by comparing our results to those of several other programs. We also present preliminary data that show SimulFold's potential as an alignment and phylogeny prediction method. SimulFold overcomes many conceptual limitations that current RNA structure prediction methods face, introduces several new theoretical techniques, and generates high-quality predictions of conserved RNA structures that may include pseudoknots. It is thus likely to have a strong impact, both on the field of RNA structure prediction and on a wide range of data analyses.

## Author Summary

Not only is the prediction of evolutionarily conserved RNA structures important for elucidating the potential functions of RNA sequences and the mechanisms by which these functions are exerted, but it also lies at the core of RNA gene prediction. To get an accurate prediction of the conserved RNA structure, we need a high-quality sequence alignment and an evolutionary tree relating several evolutionarily related sequences. These are two strong requirements that are typically difficult to fulfill unless the encoded RNA structure is already known. We present what is to our knowledge the first method that solves this chicken-and-egg problem by co-estimating all three quantities simultaneously. We show that our novel method, called SimulFold, can be successfully applied over a wide range of sequence similarities to detect conserved RNA structures, including those with pseudoknots. We also show its potential as an alignment and phylogeny prediction method. Our method overcomes several significant limitations of existing methods and has the potential to be used for a very diverse range of tasks.

## Introduction

Many RNA genes function by assuming a distinct three-dimensional structure in which the molecule folds back onto itself. Contacts are formed by hydrogen bonds between non-consecutive nucleotides that are complementary to each other. These hydrogen bonds are weak compared with covalent bonds. The three possible consensus pairs of complementary nucleotides are {A, U}, {G, C}, and {G, U}. It turns out that many properties of the three-dimensional RNA molecule can already be studied even if we know only the positions in the RNA sequence that form base-pairs. This is the level of abstraction that is predominantly chosen for studying RNA structure. For our purposes, an RNA structure is unambiguously defined by the set of base-pairing positions in the RNA sequence. This set of base-pairing sequence positions defines the RNA secondary structure. We count pseudoknotted structures, i.e., structures that contain non-nested base-pairs (e.g., two pairs *i*–*j* and *i′*–*j′* whose sequence positions are in order *i* < *i′* < *j* < *j′*) as secondary structures. The RNA structure allows us to draw conclusions about the molecule's potential function and often even the mechanism by which it acts. It is therefore of fundamental importance to be able to predict an RNA's structure from its sequence alone.

Most RNA structure prediction programs investigate only secondary structures that do not contain pseudoknots. In addition, most of the structure prediction programs aim to predict the pseudoknot-free secondary structure that minimizes the free energy of the entire RNA molecule. The first empirical and theoretical investigations of the free energies of RNA secondary structures were conducted by Tinoco and his colleges in the early 1970s [1,2]. Because the number of possible secondary structures grows exponentially with the length of the RNA sequence, algorithmic tricks have to be employed to render the calculation of the minimum free energy (MFE) secondary structure tractable. The first fast algorithm—based on a primitive scoring scheme—for finding the pseudoknot-free MFE secondary structure was proposed by Nussinov and Jacobson [3]. A few years later, Zuker and Sankoff [4] showed how similar ideas can be used to define an algorithm that calculates the pseudoknot-free MFE secondary structure using the Tinoco energy model. This algorithm still forms the basis of several of today's best MFE secondary structure prediction programs, e.g., Mfold [5–7] and the programs RNAfold and RNAalifold of the Vienna package [7–11].

The MFE approach has, however, a number of limitations. One conceptual limitation is the underlying assumption that a given RNA sequence will assume its MFE structure in the cell, i.e., its thermodynamic RNA structure. This assumption is not well supported in the general case. Theoretical, comparative studies of RNA molecules [12] show that the thermodynamic structure of even moderately long RNA molecules need not correspond to the “functional RNA structure,” i.e., the RNA structure that confers the observed functionality to the molecule and that is conserved during evolution. This may, for example, be due to co-transcriptional folding [13–15], i.e., the folding of the RNA molecule as it is being transcribed. During co-transcriptional folding, a succession of kinetic RNA structures forms along a folding pathway. None of these kinetic RNA structures needs to correspond to the MFE structure. The observed discrepancies between the observed functional structure and the predicted MFE structure may also be due to other molecules binding the RNA molecule, which can obviously influence the structure formation process. The functional secondary structure may also differ from the MFE structure by having unstructured regions that do not comprise any base-pairs. As we are interested in investigating the functional roles of RNA molecules in the cell, we therefore focus on predicting the evolutionarily conserved RNA structure rather than the thermodynamic one.

There exist several programs that aim to simulate the dynamic folding process of an RNA molecule in the cell to predict RNA structures that may contain pseudoknots [16–19]. However, these programs study only a single RNA sequence at a time, and their predictive power decreases with increasing sequence length because the error is multiplicative.

Another conceptual limitation of the MFE approach is that the Zuker–Sankoff algorithm cannot handle pseudoknotted secondary structures, i.e., structures with non-nesting base-pairs. However, pseudoknotted RNA structures are known to fulfill diverse and important functional roles in the cell [20]. We should thus aim to include them in RNA structure predictions. The prediction of pseudoknots has received more attention in the last few years, but remains very difficult. Pseudoknot prediction is, in the most general case, NP-hard even for binary strings [21,22]. For special classes of pseudoknots, structure predictions can be made more efficient from *O*(*L* ^4^) to *O*(*L* ^6^) for an RNA sequence of length *L* [23–30]. However, these algorithms are still too slow for practical purposes.

The best information for predicting the functional RNA structure can be derived from functionally equivalent RNA sequences of evolutionarily related organisms. This is due to the fact that evolutionarily related RNA sequences that serve the same purpose in the cell are likely to employ the same mechanism for exerting this function. In particular, if the function of these RNA sequences depends on their structure, this RNA structure (but not necessarily the RNA sequences themselves) should be highly conserved. If we therefore align the RNA sequences such that structurally equivalent parts are grouped together, we can detect pairs of columns in the sequence alignment where the primary sequence conservation may be low, but the functional conservation in terms of base-pairs is high. These base-pairing columns in the alignment where compensatory mutations occur in a correlated way are called co-varying or co-evolving columns. They provide the main sequence signal that many comparative structure prediction programs detect to predict the base-pairs of evolutionarily conserved RNA secondary structures. RNAalifold [10] of the Vienna package combines this type of information with a traditional MFE structure prediction, whereas Pfold [31,32] incorporates it in a score-based approach that also takes the known evolutionary relationship of the sequences in the input alignment explicitly into account. RNA-Decoder [33,34] uses an approach similar to Pfold's, but allows for extra evolutionary constraints due to known protein-coding regions in the input alignment and is the only one of the three programs capable of explicitly modeling unstructured regions. However, none of these three programs can predict pseudoknotted secondary structures.

There already exist comparative programs that attempt pseudoknot prediction. These programs use the maximum weighted matching algorithm (MWM algorithm) [35,36] to extract an RNA secondary structure that may contain pseudoknots from a given set of base-pairs with different weights. Tabaska et al. [37] use a non-comparative approach to obtain these weighted base-pairs, whereas Witwer [38] analyzes a fixed input alignment with the comparative RNA structure prediction program RNAalifold [10] (which cannot predict pseudoknotted secondary structures) to obtain weighted base-pairs. The MWM algorithm requires *O*(*L* ^3^) time to analyze an RNA sequence of length *L,* but requires a post-processing step to extract a bi-secondary structure [39]. Both programs have the same problems as the underlying algorithms and often have a low prediction accuracy [37]. Ruan et al. [40] developed a program that can utilize thermodynamic or comparative information or both. The overall accuracy is 80% for identifying base-pairs. However, this performance is achieved only with input alignments of very high quality that cannot be established without already knowing the conserved RNA secondary structure.

The fundamental conceptual problem that all of these comparative programs face is that they require an input alignment of high quality to be able to predict the conserved RNA secondary structure. However, such an alignment can often only be established if we already know the conserved RNA secondary structure. These comparative structure prediction programs thus face a major chicken-and-egg problem. If the sequences are very well conserved and easy to align based on primary sequence similarity, the resulting alignment may contain no or few co-varying columns. If, at the other extreme, the sequences are only distantly related, a trustworthy sequence alignment that would exhibit many co-varying columns is impossible to establish based on primary sequence similarity alone. Comparative RNA structure prediction methods that take a fixed alignment as input can therefore analyze only a very limited range of available data successfully.

This chicken-and-egg problem has been addressed by several comparative structure prediction programs that do not require a fixed input alignment, Dynalign [41,42], Foldalign [43,44], CARNAC [45,46], ComRNA [47], Stemloc [48], and CONSAN [49]. However, these programs find only very conserved local structures and do not model the evolutionary relationship between the sequences. ComRNA, CARNAC, and Stemloc can analyze several input sequences (Stemloc achieves this by calculating progressive pairwise alignments), whereas Dynalign, Foldalign, and CONSAN are limited to only two input sequences. ComRNA is the only one of these programs that can predict pseudoknotted secondary structures. The predictions of ComRNA rely on the calculation of maximal cliques, a problem that is known to be NP-complete. In the general case, it thus requires exponential time to run analyses, but it may be fast enough to analyze short sequences.

To summarize, all of the existing RNA structure prediction programs face at least one of the following challenges: (1) the MFE structure rather than the evolutionarily conserved structure that is likely to correspond to the functional structure is predicted, (2) unstructured regions of the RNA are not explicitly modeled, (3) input alignments are fixed and cannot be altered and improved, (4) pseudoknotted structures are either completely ignored or computationally too expensive to predict, (5) only two evolutionarily related RNA sequences are used as input, or (6) the evolutionary relationship between the RNA sequences is not explicitly modeled.

There are several good reasons to convince ourselves that many of these problems can be best solved simultaneously. For example, a good structure prediction should improve the prediction of a good alignment, and vice versa. Likewise, the prediction of a good alignment should improve the prediction of the correct evolutionary relationship of the RNA sequences, and vice versa.

The idea of co-estimating RNA secondary structures, multiple sequence alignments, and evolutionary trees was first suggested in a theory paper by David Sankoff in 1985 [50]. As the proposed strategy is computationally very demanding, this approach did not receive much attention until the mid-1990s, when Eddy and Durbin [51] and Sakakibara et al. [52] introduced covariance models (CMs). CMs employ stochastic context-free grammars (SCFGs) [52] to align a given RNA sequence to a fixed multiple sequence alignment via a consensus RNA secondary structure that may not contain pseudoknots. CMs do not explicitly model the evolution of the sequences in the alignment, but only consider different nucleic acids. Such a model can also be used for pseudoknot-free secondary structure prediction [32,34]. Modeling the insertion–deletion process with constraints on a pseudoknot-free consensus RNA secondary structure is a much harder problem, and, so far, only a few studies [53,54] have tried to address the problem. By considering both alignment and secondary structure, other studies extended the pioneering work of Sankoff without explicitly considering an evolutionary model [55,56]. As pseudoknots are context-dependent structures that cannot be modeled with SCFGs, CMs cannot be used to model pseudoknotted RNA structures.

We here propose a novel theoretical framework for solving the problem of co-estimating RNA secondary structures including pseudoknots, multiple sequence alignments, and evolutionary trees. We introduce a joint distribution of RNA structures, alignments, and trees in a Bayesian framework. As it is not feasible to analytically calculate any interesting statistics in this model in reasonable computational time, we propose a Markov chain Monte Carlo (MCMC) method with which we can sample from the posterior distribution.

## Methods

### Bayesian Considerations

According to elementary probability theory, the following equation holds:

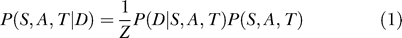
where *D* stands for data, i.e., the individual, un-aligned RNA sequences, *Z* := *P*(*D*) denotes the so-called partition function, *S* is a consensus RNA secondary structure that may contain pseudoknots, *A* is a multiple sequence alignment, and *T* is an evolutionary tree relating the sequences. Equation 1 is also called Bayes' theorem. The aim of the MCMC algorithm is to sample from the posterior probability distribution, i.e., from *P*(*S, A, T*|*D*), using the terms on the right hand side of Equation 1. For the sampling, we need to know only the ratio of the probabilities, *P*(*S*_1_, *A*_1_, *T*_1_|*D*)/*P*(*S*_2_, *A*_2_, *T*_2_|*D*). We thus have to be able to calculate *P*(*S, A, T*|*D*) and *P*(*S, A, T*) only up to a constant factor and can, for example, omit the calculation of *Z*.

### Decomposing the Posterior

We now explain how we calculate the different terms on the right hand side of Equation 1. We also introduce models and explain how to employ them to calculate the terms on the right hand side of the equation. The definitions that we propose for the prior probabilities merit a detailed discussion as there is currently no widely accepted consensus on how to define these prior distributions. Concerning the calculation of the likelihood, we make a conscious decision to use the widely known Felsenstein likelihood.

#### The likelihood.

The likelihood, *P*(*D*|*S, A, T*), is the probability of observing the individual sequences, namely *D,* as the leaves of the evolutionary tree *T* in multiple sequence alignment *A* and with the consensus RNA secondary structure *S*. We calculate the likelihood in a computationally efficient way using the Felsenstein algorithm [57], whose time requirement scales linearly with the number of sequences in the alignment and the length of the alignment. The main idea of the Felsenstein algorithm is to model the evolution of nucleotides in the alignment along the evolutionary tree *T*. For this, we partition the alignment into single, unpaired columns and pairs of base-paired columns according to the RNA secondary structure *S*. We then model the evolution along the tree with two reversible, time-continuous Markov chain models. One chain models the substitution process in every unpaired column of nucleotides involving four characters. The other chain models the substitution process of base-paired nucleotides in every pair of columns involving 16 characters [31]. For both chains, we use the same rate matrices and equilibrium distributions as the program Pfold [32]. Each of the two rate matrices, **Q**, is in diagonalized format. Transition probabilities for arbitrary evolutionary times *t* can thus be easily calculated using

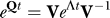
where **Λ** is a diagonal matrix containing the eigenvalues of **Q**, and **V** is the matrix containing the normalized eigenvectors of **Q**. We adhere to the computationally convenient custom [31,34,58] of treating gaps as missing information.

#### The prior.

We write the prior, *P*(*S, A, T*), as the product of the following terms:

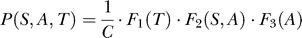
where *C* denotes an unknown normalization constant that does not depend on *S, A,* or *T*. The decomposition into the three functions *F*_1_, *F*_2_, and *F*_3_ is, to a certain extent, arbitrary and reflects our understanding of the underlying biological problem. We now explain in detail our reasons for choosing this decomposition. The consensus secondary structure, *S,* clearly depends on the alignment, *A,* e.g., its length. We cannot, for example, have a hairpin spanning 40 bases if the alignment itself is shorter than this. The alignment, *A,* is the result of an evolutionary process of insertions and deletions (indels). We describe the prior as the product of the three functions *F*_1_, *F*_2_, and *F*_3_, which we now introduce in detail.

#### The tree prior, *F*_1_(*T*).

The likelihood function, *P*(*D*|*S, A, T*), does not converge to zero for increasing branch lengths in the tree, i.e., for evolutionarily independent sequences. The integral of the likelihood function over all possible trees would therefore be infinite without a properly chosen prior for trees. We thus set *F*_1_(*T*) equal to *P*(*T*), i.e., to the prior probability for trees. One straightforward choice for the tree prior would have been Kingman's coalescent [59]; however, it assumes a molecular clock. Instead, we chose the standard exponential distribution on edge lengths as a less informative prior, 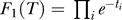 , where each *t_i_* corresponds to the length of edge *i* in the tree.

#### The structure prior, *F*_2_(*S, A*).

The consensus secondary structure, *S,* clearly depends at least on the length of the alignment, *A*. In addition, we penalize indels occurring asymmetrically in helices. Such gaps indicate bulges in some sequences, and each bulge gets a penalty of one per nucleotide. We set *F*_2_(*S, A*) equal to the prior probability of the RNA structure, *S*. By definition, the structure prior does not depend on the nucleotides in the sequences. Rather, it is the probability of observing the structure independent of the sequence itself. We use a free-energy-based model that scores the RNA structure *S* according to purely entropy-based free energies, i.e., δ*G*(*S*) = δ*S*(*S*) · *t,* where δ*S* is the entropy, which depends only on the topology of the RNA structure *S* [60], and *t* is the temperature in degrees Kelvin. *F*_2_(*S, A*) is given by

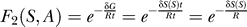
where *R* is the universal gas constant.

The entropy of a pseudoknot-free secondary structure can be calculated by decomposing it into loops [4], where the entropy contribution for each loop of length *L* is

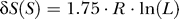



For pseudoknotted secondary structures, the calculation of the structure's entropy becomes more complicated. We use a simple model where each stretch of unpaired nucleotides of length *L* *˜* (which is neither a loop nor a stretch of sequence outside base-pairs) in a pseudoknot gets an entropy contribution of

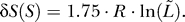



The structure prior, *F*_2_(*S, A*), therefore does not depend on the ambient temperature of the investigated RNA structures.

Other structure priors for pseudoknotted structures have, for example, been developed by Isambert and Siggia [18] and Rivas and Eddy [23,24]. While developing and testing SimulFold, we also implemented a structure prior that is equivalent to the one proposed by Rivas and Eddy, but found no improvement with respect to the prior described here (unpublished data).

#### The alignment prior, *F*_3_(*A*).

The alignment, *A,* is a result of an evolutionary indel process along the tree, *T*. We define *F*_3_(*A*) to model the gap contribution to the likelihood. We are not aware of any stochastic evolutionary model for indels that can handle an additional constraint on RNA secondary structure and that allows computationally efficient likelihood computations. We therefore use prior probabilities on alignments to incorporate indel events into our model. We choose *F*_3_(*A*) as the exponentiated penalty scores of gaps in the alignment, *A*. We decompose the alignment into homogeneous groups as shown in Figure 1. This decomposition considers only the location of gaps in the alignment and does not take the RNA secondary structure, the tree, or the different types of nucleotides in the alignment explicitly into account. Log(*F*_3_(*A*)) is the sum of terms for each column in the alignment. The contribution by each column is the sum of one or more of the following terms (which are not mutually exclusive): gap opening penalty if at least one new gap is opened in the column, gap closing penalty if at least one gap is closed in the column, and gap penalty if there is at least one gap in the column. A gap opening gets a penalty of six, a gap closing gets a penalty of six, and a gap extension gets a penalty of three. Gap opening penalties are reduced by two if there are sequences where gaps have already been opened in other alignment columns. These penalty scores are similar to the standard gap penalties commonly used in alignment programs, e.g., Clustal-X [61]. Gap opening and closing penalties are omitted at the beginning and the end of the alignment.

**Figure 1:**
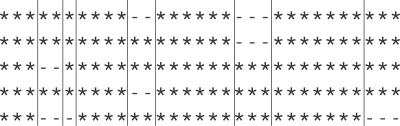
Alignment Prior For calculating the gap contribution to the prior, *F*_3_(*A*), we decompose the alignment into homogeneous groups based only on the pattern of the gaps in the alignment. Each asterisk represents a nucleotide in the alignment, and each dash denotes a gap in the alignment.

### Sampling from the Posterior Distribution

The analytical calculation of the posterior distribution is computationally too expensive. Instead, we employ a Bayesian MCMC method [62,63] to sample from the posterior distribution. This corresponds to a random walk on the possible states (*S, A, T*), whose stationary distribution is the posterior distribution, *P*(*S, A, T*|*D*). The random walk is constructed in two steps. In the first step, a new state, *X*_new_ := (*S, A, T*)_new_ in our case, is drawn from a proposal distribution, *P*, and in the second step, the discrepancy between the proposal and the target distribution, π, is corrected by accepting the proposal with probability

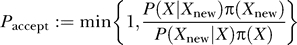
where *X* = (*S, A, T*) is the actual state of the chain. The chain remains in state *X* with probability 1 − *P*_accept_ [62,64].

The mixing of the Markov chain depends on how closely the proposal distribution resembles the target distribution. Gibbs sampling is a special case of MCMC sampling, where each state *X* can be described as a multidimensional vector ***X*** = (*x*_1_, *x*_2_,...*x_n_*) and where it is possible to draw a new random coordinate *x_i_* from the conditional distribution *P*(*x_i_*|*X*[−*i*]) for any state *X* and any coordinate *i*. ***X***[−*i*] denotes the vector of coordinates without coordinate *i*. As the newly drawn coordinate is always accepted, the Gibbs sampler is an MCMC method with an acceptance probability of one.

As it is generally not possible to sample from an arbitrary conditional distribution, the Gibbs sampling strategy can only rarely be used. However, it is possible to mimic the conditional distribution with an auxiliary distribution. This strategy is employed in partial importance sampling; see MacKay [65] for an overview of different sampling strategies. Importance sampling has been successfully used to model different distributions that occur in the context of bioinformatics [66,67], and we employ it for proposing alignments and RNA secondary structures. We define a Markov chain that converges to the desired distribution and then use this chain to sample from the posterior. As we have seen in the section before, the posterior distribution is a joint distribution on RNA structures, multiple sequence alignments, and trees. The challenge is to define moves on this joint distribution that are reversible and ergodic, and that satisfy detailed balance [63].

It is possible to define tree moves that are independent from the actual alignment and RNA structure. However, it is generally impossible to alter the alignment without disturbing the RNA structure. We therefore use the following three types of moves: changing the length of an edge in the tree, changing the tree topology, and using a complex move that alters both the RNA structure and the alignment.

#### Tree moves and tree sampling.

We use the Metropolis-Hastings algorithm [64] for sampling tree moves. The tree sampling is divided into two steps, one to change the length of an edge and one to change the tree's topology. We use standard moves for changing the edge lengths [68], and nearest neighbour interchange [69,70] for changing the tree topology. For sampling the edge length, we first pick an edge of the tree at random and then choose a new edge length, *L*_new_, from the interval [max{0, *L −* δ, *L* + δ}] where *L* is the old edge length and δ := 0.1, the fixed span for sampling edge lengths. Using the Felsenstein algorithm [57], we then calculate the loglikelihood of the alignment given the old tree, log(*P*(*D*|*S, A, T*)), and the loglikelihood of the alignment given the new tree with the new edge length, log(*P*(*D*|*S, A, T*_new_)). We accept the new edge length if a random number *r* ∈ [0, 1] is smaller than the following Metropolis-Hastings ratio, i.e., if

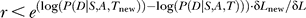
where δ*L*_new_ is the length of interval [max {0, *L*_new_ − δ}, *L*_new_ + δ] and δ*L* is the length of interval [max {0, *L* − δ}, *L* + δ], and we reject the new edge length otherwise.

For changing the topology of the tree, we pick a tree node at random and swap this node and its aunt node to alter its topology (see Figure 2). These moves have been shown [68,71] to be ergodic, i.e., any tree topology can be transformed into any other tree topology using these moves. We then calculate the loglikelihood of the alignment given the old tree, log(*P*(*D*|*S, A, T*)), and the loglikelihood of the alignment given the new tree with the new tree topology, log(*P*(*D*|*S, A, T*_new_)). We accept the new tree topology if a random number *r* ∈ [0, 1] is smaller than the following Metropolis-Hastings ratio, i.e., if

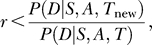
and reject the new tree topology otherwise.

**Figure 2:**
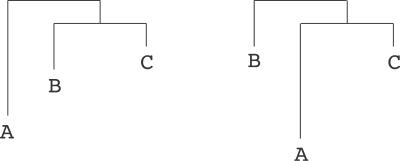
Sampling of Tree Topologies The topology of the tree on the left gets modified into the tree on the right by swapping an aunt (A) and its niece (B). Nice–aunt swapping has been shown to be ergodic.

#### Structure and alignment moves and sampling.

Simultaneously changing the structure and alignment is more sophisticated and merits a detailed discussion. The challenge is to define moves that can significantly change the actual state, that can be calculated efficiently, and that have a high probability of being accepted. Figure 3 gives a symbolic description of our sampling strategy.

**Figure 3:**
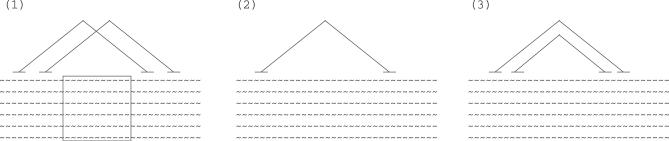
Sampling Alignment and Structure We sample a new alignment by choosing a random window of the alignment that is devoid of base-pairs and by realigning it (step 1). We then sample a new RNA structure by removing a random set of helices from the given structure (step 2) and by adding a set of new helices (step 3).

The alignment is sampled in the following way while keeping the RNA structure and tree fixed. We first find intervals in the alignment that do not contain any base-paired positions and that cannot be further extended. We then choose one of these intervals at random and propose a new alignment for this interval. This alignment is created using a stochastic version of iterative alignment along the fixed tree [67], where two alignments are merged at each internal tree node into a new one by forward–backward sampling from the posterior of a pair hidden Markov model [72]. The resulting new overall alignment is the proposal alignment. We accept it if the following Metropolis-Hastings ratio is larger than a random number *r* ∈ [0, 1]:

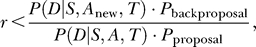
and reject it otherwise. *P*(*D*|*S, A*_new_, *T*) denotes the likelihood given the new alignment and *P*(*D*|*S, A, T*) the likelihood given the old alignment, calculated as described above. *P*_proposal_ and *P*_backproposal_ denote the proposal and backproposal probabilities, respectively. The backproposal probability is defined as the probability of choosing the old state from the proposal distribution given that we are in the new state of the Markov chain.

Once the alignment has been sampled, we sample the RNA structure while keeping the alignment and the tree fixed. The challenge is to devise a unique way of proposing a new structure; otherwise we cannot easily calculate the proposal and backproposal probabilities. We propose a new structure in the following way. We first decide on the number of helices to be removed from the given set of helices by drawing a random number from the truncated Poisson distribution with parameter λ = 3. We then remove this number of helices from a weighted distribution, where the weight of each helix is the log-odds ratio of its Felsenstein likelihoods (i.e., the likelihood of the RNA without any base-pairs and the likelihood of the RNA with the helix). The set of removed helices is denoted *R*. We then propose a new set of helices, *N*. Before starting the MCMC, we calculate two matrices for each RNA sequence in *D,* denoted **H** and **E**, which remain unchanged during the MCMC run. **H** is a two-dimensional matrix where matrix element *H*(*i, j*) is the score of the best helix whose outer base-pair is at sequence positions {*i, j*}. The elements in the matrix are calculated using dynamic programming. ***E*** is a one-dimensional vector where entry *E*(*i*) = Σ*_j_ H*(*i, j*) is the score for starting a helix at sequence position *i* on the 5′ side. Each new helix in set *N* is sampled in the following way. We scan the alignment from left to right and make a random decision where to start the helix. We choose the 5′ start of the new helix proportional to the sum of *E* values in that column of the alignment, i.e., we use importance sampling. Once the 5′ start of the new helix has been fixed, we choose the 3′ end of the helix proportional to the sum of *H* values in the corresponding column of the alignment. Now that both ends of the new helix are fixed, we choose the number of consecutive base-pairs in the helix based on the quality of the resulting helix. The quality of a helix is defined as the log-odds ratio of its Felsenstein likelihoods (i.e., the likelihood of the RNA without the helix and the likelihood of the RNA with the helix). Using this procedure, we can sample a reasonable new helix in a computationally very efficient way that requires only linear rather than cubic time. We accept the new RNA structure that differs from the old RNA structure by the set of removed helices, *N,* if the following Metropolis-Hastings ratio is larger than a random number *r* ∈ [0, 1]:

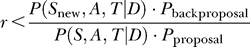
where *P*(*S*_new_, *A, T*|*D*) is the posterior probability given the new RNA structure and *P*_proposal_ and *P*_backproposal_ are the proposal and backproposal probabilities, respectively.

### Analyzing the MCMC Results

The primary result of an MCMC run is a large set of simulated (*S, T, A*) triples that are distributed according to the posterior distribution. This data needs post-processing to characterize and visualize the posterior distribution. As we are interested in deriving the RNA structure that is best supported by the posterior distribution, we therefore marginalize with respect to the RNA structure.

We project the RNA structure, *S,* of each sampled (*S, T, A*) triple onto the RNA sequences in *D* and thereby obtain a set of RNA structures for each individual RNA sequence. The challenge is to combine these structures into a single RNA structure that captures the prominent features of the set of structures.

There already exist a number of programs that determine a consensus structure for a given set of RNA structures, e.g., RNAdistance of the Vienna package [7,73] and RNA-Forester [74,75]. RNA-Forester computes a global structural alignment for several unaligned sequences with known secondary structures using a dynamic programming procedure that depends on scores with combined information on structure and sequence similarity. However, RNA-Forester and RNAdistance both require the input RNA structures to be secondary structures and cannot handle pseudoknots.

#### Deriving a consensus RNA structure including pseudoknots.

We use the following procedure for deriving a consensus bi-secondary structure for each individual sequence. For each simulated (*S, T, A*) triple, we project the RNA structure, *S,* onto the individual sequences in *D*. For each RNA sequence, we then transform the set of its structures into a table of base-pairing probabilities by converting the relative frequency of base-pairs in the RNA structures into estimates of base-pairing probabilities. The set of base-pairs with pairing probabilities larger than zero is then used as input to the MWM algorithm [35,36]. We use the algorithm in its implementation by Rothberg [76], which requires *O*(*L* ^3^) time and *O*(*L* ^2^) memory to analyze a sequence in which *L* positions can be base-paired. The MWM algorithm operates on an undirected, weighted graph where each node represents a sequence position and each edge between two nodes represents a base-pair between the two corresponding sequence positions. We set the weight of each edge to the weight of the helix to which this base-pair belongs, and define the weight of a helix as the sum of the probabilities of its base-pairs. The MWM algorithm determines the highest-scoring subset of mutually compatible base-pairs that, in mathematical terms, corresponds to a maximum weighted matching in the graph. Two base-pairs are defined as compatible if they do not share a sequence position. In the general case, the base-pairs of the maximum weighted matching need not correspond to a bi-secondary structure. Bi-secondary structures [39] are planar structures that can be visualized as the superposition of at most two disjoint secondary structures. The class of bi-secondary structures comprises secondary structures and a wide variety of pseudoknots, but excludes true knots. The α-operon mRNA structure is one of the very few examples that is not a bi-secondary structure; refer to Condon et al. [77] for an overview of the different classes of pseudoknots and their relationships to each other. As there is yet no variant of the MWM algorithm with a constraint on bi-secondary structures, we need a post-processing step to extract a high-scoring bi-secondary structure from the base-pairs of the maximum weighted matching. We do this in the following way. We order the helices by decreasing weight and assign colour 1 to the first and highest-scoring helix. The next uncoloured helix in the list gets the same colour as the first set of already coloured helices with which it can form a secondary structure (the sets of already coloured helices are ordered by increasing colour-number). If the uncoloured helix cannot form a valid secondary structure with any set of already coloured helices, it is assigned to the next available new colour. Once all helices have been coloured, the helices of colours 1 and 2 (if any helices of colour 2 exist) form the final bi-secondary structure. This RNA structure is the RNA structure that is predicted by SimulFold for that individual RNA sequence. We implemented the structure post-processing step in a dedicated program, called bp2bistruc, which takes an upper-triangle matrix of base-pairing probabilities as input and calculates a high-scoring bi-secondary RNA structure. This predicted structure is accompanied by posterior probabilities, which are useful to highlight the more and the less reliably estimated parts of the predicted structure (see Figure 4).

**Figure 4:**
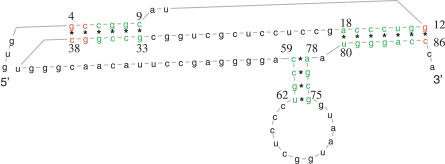
RNA Structure with Pseudoknot Predicted by SimulFold for the HDV Sequence of Organism AJ309880 Using the MCMC Results Generated with Parallel Tempering The pairing probabilities estimated by SimulFold are colour-coded and range from bright green (high probability) to bright red (low probability). The pairing probabilities for this structure range from 0.62 for the pair at sequence positions {4, 38} to one for most of the base-pairs, e.g., the one at sequence positions {6, 36}. For this figure, we have adopted a nonlinear colouring scheme; otherwise, all base-pairs would simply come in slightly different shades of green.

## Results

### Features of Available Secondary Structure Prediction Programs

SimulFold is to our knowledge the first program that predicts an RNA structure including pseudoknots while simultaneously estimating an alignment as well as an evolutionary tree for several, evolutionarily related input RNA sequences. It was therefore not possible to present a comparison to a truly equivalent program. Instead, we compare the RNA structures predicted by SimulFold to those predicted by RNAalifold [10], Hxmatch [78], Pfold [31,32], and CARNAC [45,46].

RNAalifold takes a fixed alignment as input and predicts a consensus RNA secondary structure without pseudoknots. It extends the MFE algorithm employed by the non-comparative MFE methods Mfold and RNAfold by interpreting the fixed input alignment as a hyper-sequence and by simultaneously minimizing the overall free energy while taking the primary sequence conservation and co-varying columns in the fixed alignment into account. The optimization is implemented in a dynamic programming procedure that combines free energy parameters with conservation scores.

Hxmatch is an extension of RNAalifold. Like RNAalifold, it takes a fixed alignment as the only input and predicts a consensus RNA secondary structure. However, unlike RNAalifold, it is capable of predicting secondary structures with pseudoknots. Hxmatch employs a two-step procedure. In the first step, the fixed input alignment is analyzed with RNAalifold [10] of the Vienna package, which calculates the base-pairing probability for each possible pair of columns in the alignment by considering all possible secondary structures without pseudoknots. In the second step, these weighted base-pairs are used as input to the MWM algorithm. The MWM algorithm derives the highest-scoring subset of mutually compatible base-pairs, requiring *O*(*L* ^3^) time and *O*(*L* ^2^) memory to analyze an input alignment of length *L*. As these base-pairs need not correspond to a bi-secondary structure, a heuristic, greedy algorithm is then employed to extract a bi-secondary structure. The MWM algorithm and the greedy algorithm are repeatedly used until the resulting bi-secondary structure remains unchanged or 30 iterations have been completed. Hxmatch does not take the evolutionary relationship of the input RNA sequences explicitly into account.

Pfold takes as input not only a fixed alignment, but also an evolutionary tree relating the sequences, and predicts a consensus secondary structure which does not contain pseudoknots, as Pfold cannot handle pseudoknots. Pfold employs an SCFG, i.e., a probabilistic rather than an MFE model, to derive the consensus secondary structure. Similar to RNAalifold, it takes the primary sequence conservation and co-varying columns in the fixed input alignment into account. Unlike RNAalifold, Pfold also takes the known evolutionary relationship of the input sequences, i.e., the input tree, explicitly into account. Both, Pfold, and RNAalifold require *O*(*L* ^3^) time and *O*(*L* ^2^) memory to analyze an input alignment of length *L*.

CARNAC is also a comparative RNA structure prediction method. It takes several unaligned RNA sequences as input and predicts an RNA structure for each individual RNA sequence which does not contain pseudoknots, as CARNAC cannot handle pseudoknots. Similarly to Hxmatch and RNAalifold, it does not take the evolutionary relationship of the input sequences explicitly into account. CARNAC employs a multi-step procedure for generating predictions. In the first step, potential helices are predicted for each RNA sequence separately. In the second step, an optimal consensus secondary structure is extracted from these helices for every possible pair of RNA sequences. In the third and last step, the different secondary structures that were predicted in a pairwise fashion for each individual RNA sequence are combined into one secondary structure using graph theoretical techniques. This is the final RNA structure reported by CARNAC for that RNA sequence. In the most general case, the algorithms underlying CARNAC would require *O*(*L* ^6^) time and *O*(*L* ^4^) memory to analyze input sequences of length *L* [45]. These requirements can be reduced by a number of computational tricks. For the data investigated by Perriquet et al. [45], the empirically observed requirements were approximately *O*(*L* ^2^) time and memory.

### Dataset

We compiled a large and diverse dataset from previously published data [78–80] to thoroughly investigate SimulFold's ability to correctly predict RNA structures. Our dataset consists of 16 sets of evolutionarily related sequences that cover a wide range of average pairwise sequence identities (pids) and sequence lengths. Half of the sets contain a pseudoknotted reference structure; the other half contain a reference structure without pseudoknots. The number of sequences in each set ranges from five to 15 sequences. We automatically generated Clustal-W alignments for all 16 sets to serve as input alignment for those programs that take a fixed input alignment. The resulting alignments are 74–1,601 nucleotides long, and their average pid ranges from 40% to 91%. Table 1 summarizes the main characteristics of each set (see the columns “Structure” and “Alignment” and the caption of the table).

**Table 1:**
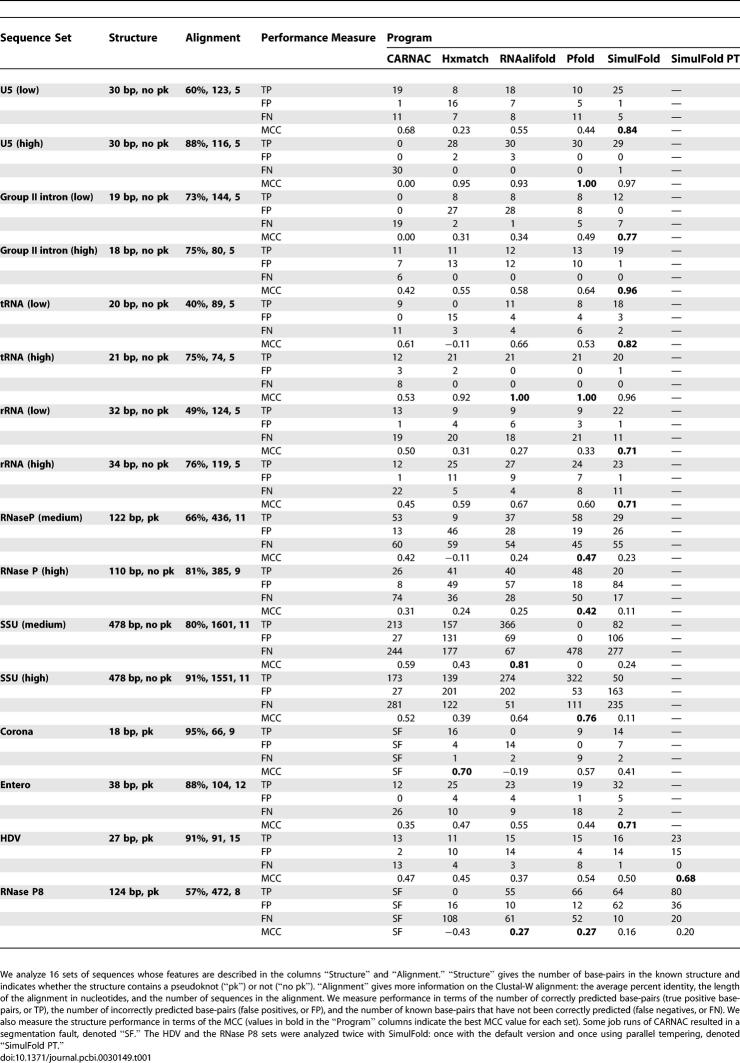
Performance of CARNAC, Hxmatch, RNAalifold, Pfold, and SimulFold for Predicting RNA Structures

### Performance Evaluation

Table 1 shows the performance values for the RNA structures predicted by SimulFold, RNAalifold, Hxmatch, Pfold, and CARNAC for all 16 sets. We used the Clustal-W alignments as fixed input alignments for Hxmatch and Pfold. The same alignments were also used as initial alignments for SimulFold.

To evaluate the structure prediction performance, we compared the known RNA structure of the reference organism in each set with the corresponding predicted RNA structure. We measured the quality of the structure predictions in terms of the number of correctly predicted base-pairs (true positive base-pairs, or TP, see Table 1), the number of incorrectly predicted base-pairs (false positives, or FP), and the number of known base-pairs that have not been correctly predicted (false negatives, or FN). We also calculated Mathews's correlation coefficient (MCC) (see Table 1), which is defined as

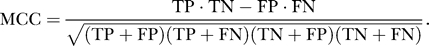



CARNAC generally shows a high specificity, i.e., a low number of incorrectly predicted base-pairs, often in combination with a low sensitivity, i.e., a low number of true positive base-pairs. This low sensitivity can even be found for sets whose average pid is fairly high, e.g., set U5 (high) with an average pid of 88%. CARNAC's performance is naturally limited by the fact that it cannot predict pseudoknotted structures.

Besides SimulFold, Hxmatch is the only other investigated program that is capable of predicting pseudoknotted secondary structures. Hxmatch has the tendency to over-predict base-pairs, as indicated by the high number of false positive base-pairs. This happens for low average pids (e.g., set tRNA [low]) and for high pids (e.g., set SSU [high]). It is interesting to note that RNAalifold often does better than Hxmatch at predicting the base-pairs of pseudoknotted reference structures, e.g., the results for RNaseP (medium), RNase P8, SSU (medium), and SSU (high). However, there are also examples, see the corona set, where the reverse holds.

Like RNAalifold and Hxmatch, Pfold is a program that takes a fixed alignment as input. Its performance tends to be low for the low average pid range, e.g., the U5 (low), tRNA (low), and rRNA (low) sets, which all have average pids below 50%. For the high pid range, its performance can be limited because of the fact that Pfold cannot model pseudoknots, e.g., in the corona, entero, and hepatitis delta virus (HDV) sets. Its performance for the RNaseP (medium) set constitutes a notable exception to this trend.

SimulFold is the only program that simultaneously co-estimates alignments, structures, and trees. It clearly outperforms all other programs in terms of overall performance for eight out of 16 sets: U5 (low), group II intron (low and high), tRNA (low), rRNA (low and high), entero, and HDV. It also shows a competitive performance for the sets U5 (high) and tRNA (high). These sets cover a wide range of average pids, from 40% to 91%. The results for the two SSU sets show that SimulFold has problems analyzing these two sets, whose reference alignments span more than 1,500 nucleotides. However, the results for the RNase P8 set show that SimulFold can successfully predict structures with high sensitivity even for comparatively long sequences (the reference alignment of the RNase P8 set has a length of 472 nucleotides). The results for the RNase P8 and the HDV sets show the benefits of parallel tempering. When investigating the predictions for the HDV set, we concluded from the loglikelihood plot (see Figure 5) that the MCMC chain got stuck in local minima. We therefore implemented a more sophisticated version of SimulFold that employs the MCMC technique of parallel tempering [81] to address the problem. As the grey line in Figure 5 shows, parallel tempering solves the mixing problem for the HDV set and significantly improved the sensitivity, while at the same time reducing the number of incorrectly predicted base-pairs.

**Figure 5:**
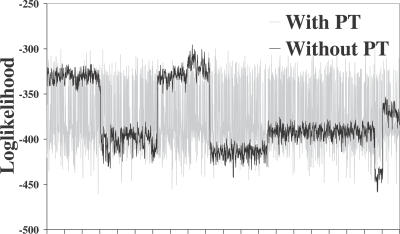
Loglikelihood as a Function of the Steps in the MCMC Chain for the HDV set with and without Parallel Tempering With parallel tempering (grey) and without parallel tempering (black). Without parallel tempering, the MCMC chain gets stuck in local minima.

### Potential of SimulFold as an Alignment and Phylogeny Prediction Program

Our initial motivation for devising a novel method that simultaneously co-estimates RNA structures, alignments, and evolutionary trees was to improve the prediction of RNA structures, in particular those with pseudoknots. A very interesting additional benefit of our approach is that SimulFold can also be used as an alignment and phylogeny prediction program.

We here present preliminary results for sequences from the HDV set that show SimulFold's potential as an alignment and phylogeny prediction program. By the same argument that we made above for RNA structure prediction, we should also be able to derive better alignments and trees if we co-estimate all three, interdependent quantities together rather than in isolation.

The HDV dataset contains 15 sequences of HDV ribozymes from several strains (their NCBI accession numbers are shown on the figures showing the alignments and the consensus networks). The ribozyme contains one pseudoknot and a variable helix. We calculated posterior probabilities of alignment columns from the multiple alignments that the MCMC method sampled, i.e., the probability of seeing a particular alignment column in a sampled multiple alignment.

We calculated the maximum posterior decoding (MPD) alignment using a dynamic programming procedure [72]. It has been shown [67] that MPD yields better estimates than maximum a posteriori alignment estimation from an MCMC sample.

The MPD alignment is shown in Figure 6, together with the estimated posterior probabilities for each column as well as the reference secondary structure that includes a pseudoknot. The figure clearly highlights three regions in the alignment where lower posterior probabilities are due to an ambiguity in the estimation. The first region overlaps a hairpin loop. Even though the MPD alignment contains no gaps in this region, the sequences vary a lot and there exist several plausible explanations that relate the sequences in this region in terms of evolutionary indel events. The remaining two regions overlap the two base-paired sides of a variable helix. The low posterior probabilities indicate that several plausible alignments exist for these regions. These observations are in line with our difficulty to correctly predict these parts of this helix. We conjecture that this helix may be shorter or contain bulges in some of the sequences of the HDV set.

**Figure 6:**
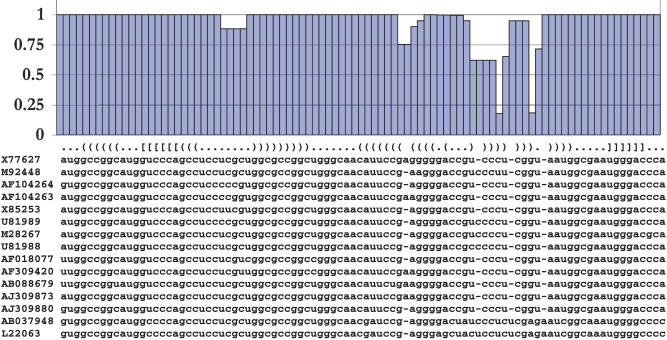
MPD Alignment for the HDV Dataset Consisting of 15 Sequences of HDV Ribozymes The name of each sequence indicates the NCBI accession number of the strain. The ribozyme contains one pseudoknot and a variable helix as shown in the line above the alignment, which denotes the known reference structure in dot-bracket, or Vienna, notation. The posterior probabilities for each alignment column were derived from the multiple sequence alignments that the MCMC method sampled and are indicated at the top of the figure.

If we compare our MPD alignment to the alignment generated by Clustal-X [61] shown in Figure 7, we observe two main differences. First, Clustal-X does not take secondary structure into account when predicting the alignment. This yields several nonsense base-pairs with respect to the known reference structure (highlighted in green in Figure 7). Second, Clustal-X does not evaluate the reliability of the different regions in the predicted alignment. We thus do not know which parts of the predicted alignment are particularly well or poorly supported by the data.

**Figure 7:**
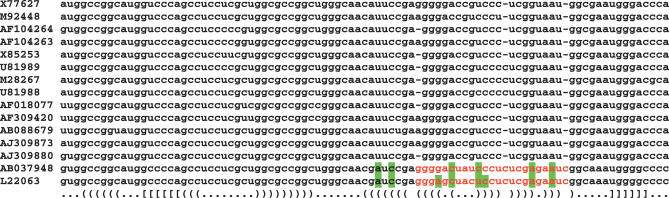
Clustal-X Alignment for the HDV Dataset Consisting of 15 Sequences of HDV Ribozymes Some parts of the AB037948 and L22063 sequences are misaligned (red characters), which causes nonsense base-pairs (highlighted in green) when mapping the known reference structure onto these sequences.

We calculated consensus networks based on the evolutionary trees sampled from the posterior distribution by the MCMC using the method of Holland and Moulton [82] implemented in the SplitsTree4 program [83]. We set the threshold for splits to 0.1, i.e., we retained only splits that were present in at least 10% of the sampled trees, and generated the two networks shown in Figure 8. The two networks have the same topology, but differ in the lengths of their edges, which represent different kinds of information. In the left network, the length of each edge is proportional to the probability of the split that is represented by the edge in the posterior distribution (the unit in the top left corner shows 1,000 occurrences in 2,000 sampled trees). In the right network, the length of each edge is equal to the average length of the edge in the sampled trees that contain that edge. There are five groups of strains: the lone strain AJ309873; a group containing U81988, M28267, X77627, and M92448; another group containing U81989, AF104263, AF104264, and X85253; and finally two relatively close groups containing AB088679, AF018077, and AF309420, and L22063, AB03748, and AJ309880. There is not enough phylogenetic signal to infer the relationship between the union of the two last groups and the other three groups. As Figure 8 indicates, there are several plausible explanations for how strains in the first two groups could have evolved.

**Figure 8:**
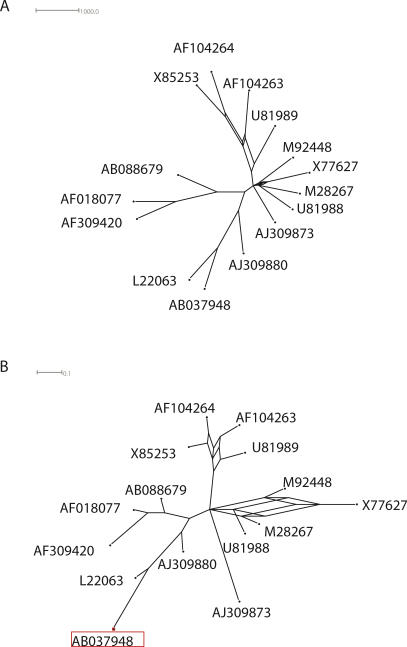
Two Consensus Networks for the HDV Dataset Consisting of 15 Sequences of HDV Ribozymes The name of each sequence indicates the NCBI accession number of the strain (see also Figure 6)**.** The edge lengths in the left network correspond to the probability of the corresponding split in the posterior distribution, whereas the edge lengths in the right network correspond to the average length of the edge in the sampled trees.

These preliminary results show that SimulFold not only allows us to derive consensus multiple alignments and evolutionary trees, but even enables us to highlight particularly well or poorly estimated parts of these alignments and trees.

It is easy to think of situations where one does not want to simultaneously co-estimate RNA structures, alignments, and trees, e.g., because a high-confidence RNA structure (or alignment or tree) has already been established. It is straightforward to employ SimulFold in these situations, as the program can be easily told to keep the input RNA structure or alignment or tree (or any combination thereof) fixed.

### Evaluation of the MCMC's Efficiency

An MCMC can suffer from a low efficiency for three main reasons: (1) the acceptance ratio is low, (2) the Markov chain gets stuck in local optima, or (3) the computational time to perform each step is large. We introduced partial Metropolis importance sampling to quickly propose moves that replace only part of the data and to keep the rejection probability and autocorrelation low. For the HDV set, the initial loglikelihood plot shows poor mixing (see Figure 5). We therefore implemented the more sophisticated MCMC technique of parallel tempering in SimulFold. This option can be switched on whenever the mixing properties need to be improved. Figure 5 shows, for the HDV set, how parallel tempering can considerably improve the mixing properties. The parallel tempering run took 1.5 d and used 50 MB of memory on an Intel Xeon dual 3 GHz machine for the HDV set, involving seven parallel chains, 10,000 steps for burn-in, and 2,000 samples, and making 100 steps between two samplings. Shorter sequences, multiple alignments with fewer sequences, and MCMC runs without parallel chains took proportionally less time. In terms of computational complexity, SimulFold takes *O*(*N* · *L*) time to propose a change in phylogeny for *N* sequences and an alignment of length *L*. Changing the alignment takes *O*(*N* · *W* ^2^) time, where *W* is the length of the window that is re-sampled. Changing the structure takes *O*(*K* · *N* · *L*) time, where *K* is the number of changed helices. In its current implementation, SimulFold uses the same number of moves to update trees, alignments, and structures. The mixing of the chain decreases with the length and number of sequences. We did not investigate this behaviour in detail, but know that for multiple sequence alignments, the mixing time is proportional to *N* · *L*.

## Discussion

We propose here a novel theoretical framework for co-estimating an RNA structure including pseudoknots, *S,* a multiple-sequence alignment, *A,* and an evolutionary tree, *T,* given several evolutionarily related RNA sequences, *D,* as input. We also present an implementation of this framework in a new computer program, called SimulFold, and evaluate the quality of the predicted RNA structures relative to those predicted by existing programs.

Our novel theoretical framework allows us to sample (*S, T, A*) triples from the posterior distribution, *P*(*S, A, T*|*D*), in a computationally very efficient way using a Bayesian MCMC. For every RNA sequence in *D,* we then extract the RNA structure that is best supported by the posterior distribution using the MWM algorithm and a post-processing step implemented in an auxiliary program called bp2bistruc.

Our work is significant in a number of ways. SimulFold overcomes several limitations of existing RNA structure prediction methods, in particular the conceptual limitations of SCFG-based methods. SimulFold does not rely on a fixed input alignment or tree, it can predict pseudoknotted RNA structures, it can take any number of related RNA sequences as input, it aims to predict the evolutionarily conserved RNA structure rather than the thermodynamic or MFE structure, it explicitly models the evolutionary relationship between the RNA sequences, it is a fully probabilistic method that is capable of quantifying the reliability of its predictions, and, most important for the majority of users, it works in a computationally efficient way and can be used on any standard desktop computer. Furthermore, SimulFold derives the RNA structure that is best supported by the posterior distribution, rather than the RNA structure that maximizes the likelihood, which is what SCFG-based structure prediction methods do.

We use a number of novel theoretical and computational tricks to achieve the above. We devised a new expression for the prior *P*(*S, A, T*), in particular a function that models the contribution of trees, a function that incorporates information on structures and alignments, and a function that quantifies the contribution of gaps in the alignment. For sampling from the posterior distribution, we propose a new way of sampling trees and a fairly sophisticated new way of jointly sampling structures and alignments in a computationally very efficient way. After *O*(*N* · *L* ^2^) pre-processing time, we do an MCMC step modifying the base-pairs that requires *O*(*N* · *L*) time, where *N* is the number of sequences and *L* is the average length of the sequences. We also introduce a new type of MCMC sampler that we call a partial Metropolis importance sampler. We implemented the sophisticated MCMC technique of parallel tempering into SimulFold, which can be switched on whenever the loglikelihood plot indicates poor mixing properties. Finally, we introduce a new program, bp2bistruc, that derives an RNA structure that may include pseudoknots (a bi-secondary structure, to be precise) from an input table of base-pairing probabilities.

The performance of SimulFold in predicting RNA secondary structures with and without pseudoknots compares very well to the performance of RNAalifold, Hxmatch, Pfold, and CARNAC across a wide range of average pids and sequence lengths. We also present encouraging preliminary results that show SimulFold's potential as an alignment and phylogeny prediction program. It is not only possible to derive a consensus alignment and tree, but also to highlight those parts of the alignment and tree that can be particularly well or poorly estimated. This information is very valuable for interpreting the results in great detail.

It is easy to think of situations where one does not want to simultaneously co-estimate RNA structures, alignments, and trees, e.g., because a high-confidence RNA structure (or alignment or tree) has already been established. We therefore implemented special flags in SimulFold that allow the user to keep the input RNA structure or alignment or tree (or any combination thereof) fixed. We hope that this feature will make SimulFold a useful program for a wide range of interesting tasks and data analyses.

In the future, we intend to investigate different models and priors for use in SimulFold, e.g., a co-transcriptional folding prior. We also hope to further improve the properties of the sampling, e.g., partial importance sampling of tree or an even better structure sampler, to improve the performance for very long sequences.

SimulFold opens up a large number of possibilities for exciting data analysis. Most importantly, we can now start analyzing data whose low primary sequence conservation has so far prevented their analysis with methods that require a high-quality input alignment. We hope that our work inspires other researchers to also develop methods that predict or investigate the functional structure of RNA sequences so that we learn more about how RNA sequences play their diverse functional roles in the cell.

SimulFold as well as information on the input and output files of this analysis can be found at http://www.cs.ubc.ca/∼irmtraud/simulfold/.

## Abbreviations

CM: covariance model
FN: false negative
FP: false positive
HDV: hepatitis delta virus
indel;: insertion and deletion
MCC: Mathews's correlation coefficient
MCMC: Markov chain Monte Carlo
MFE: minimum free energy
MPD: maximum posterior decoding
MWM algorithm: maximum weighted matching algorithm
pid: pairwise sequence identity
SCFG: stochastic context-free grammar
TP: true positive
